# *IUCrJ* on passing its tenth anniversary and entering its second decade: progress, current status and prospects for the future

**DOI:** 10.1107/S205225252401217X

**Published:** 2025-01-01

**Authors:** Andrew. J. Allen

**Affiliations:** a Materials Measurement Science Division National Institute of Standards and Technology 100 Bureau Drive Gaithersburg MD 20899-8520 USA

**Keywords:** *IUCrJ*, IUCr Journals, International Union of Crystallography

## Abstract

This Editorial briefly celebrates the history and progress of *IUCrJ* in its first decade, reviews its present status, and suggests some pointers for the future.

*IUCrJ* was launched in 2014 under my predecessor as Editor-in-Chief of IUCr Journals, Samar Hasnain. Its launch coincided with the UNESCO International Year of Crystallography, some hundred years after the seminal discovery of X-ray diffraction by Max von Laue and the Braggs, father and son, and in the centennial year of the award of the Nobel Prize to Max von Laue. As was pointed out in its inaugural Editorial (Hasnain, 2014 ▸), *IUCrJ* was launched as a fully open-access IUCr flagship journal with the objective of attracting high-quality science papers of broad scientific significance from across all the scientific communities that use results obtained from diffraction methods. Over the years since 2014, *IUCrJ* has provided a home for many highly cited papers across a broad area of structural science and crystallography, and brief highlights of several representative papers published in *IUCrJ* were included in the IUCr’s 75th Anniversary Editorial (Allen, 2023 ▸).

Initially papers were welcomed in five main research areas: *Biology and Medicine*, *Chemistry and Crystal Engineering*, *Materials and Computation*, *Neutron and Synchrotron Science and Technology*, and *Physics and Free-Electron Laser Science and Technology*. In 2016, *cryoEM* was added and, from 2022, *Electron Crystallography*. Within many papers there is significant overlap among these areas. From the beginning, many *IUCrJ* articles have proved to be of high impact and been well cited or, in the case of recent articles, clearly show potential for high impact. The types of articles published range from short *Research Letters* of immediate interest (*e.g.* Joosten *et al.*, 2014 ▸; Stellato *et al.*, 2014 ▸; Kidmose *et al.*, 2019 ▸, Broadhurst *et al.*, 2021 ▸; Chodkiewicz *et al.*, 2024 ▸), to concise *Research Papers* (*e.g.* Jelsch *et al.*, 2014 ▸; Tria *et al.*, 2015 ▸; Hoshino *et al.*, 2016 ▸; Mackenzie *et al.*, 2017 ▸; Park *et al.*, 2017 ▸; Zivanov *et al.*, 2019 ▸; Liu *et al.*, 2022 ▸), as well as up-to-date *Topical Reviews* (*e.g.* Blakeley *et al.*, 2015 ▸; Bryce, 2017 ▸; Clabbers *et al.*, 2022 ▸; Burley *et al.*, 2024 ▸) and in-depth *Feature Articles* (*e.g.* Schlichting, 2015 ▸; Hexemer & Müller-Buschbaum, 2015 ▸; Spence, 2017 ▸; Loch & Jaskolski, 2021 ▸; Brink *et al.*, 2024 ▸), with occasional *Lead Articles* (*e.g.* Gagné & Hawthorne, 2020 ▸; McNulty & Lightfoot, 2021 ▸). *Editorials* are published in most issues and focus on a different area of structural science each time. *Scientific Commentaries* on articles appearing in the same or recent issues appear regularly. Some of the Editorials and Commentaries have attracted, or show promise in attracting, significant numbers of citations themselves (*e.g.* Metrangolo & Resnati, 2014 ▸; Subramaniam *et al.*, 2016 ▸; Chapman, 2023 ▸; Bourne, 2024 ▸).

Key attributes of articles appearing in *IUCrJ* are that they should be ‘cross-cutting’ and ‘high impact’. While there will always be a desire for any given *IUCrJ* paper to have both these attributes, a cross-cutting paper (across different aspects of structural science) is expected to have significant impact in one or more of those fields, and a high-impact paper, even in just one field, is expected to include concise introductory material to allow a broader audience to appreciate the impact of what has been accomplished. With these points in mind, a covering letter is required with each regular submission to *IUCrJ*, other than Editorials and Commentaries, and a vote is taken among the Main Editors (each leading one of its research subject areas) to determine whether the article should be assigned to a Co-editor and sent out for review. If there is a tie in voting, the Editor-in-Chief is automatically asked to intervene to make a decision. In so doing, attention is paid to the vote and any comment of the Main Editor(s) most closely connected with the subject area of the paper and these may be sought if not previously provided before a final decision is made. This procedure generally sets the scene for a timely but thorough review of high-quality submissions to *IUCrJ* that have passed through pre-selection. Meanwhile, high-quality articles found to be too specialized to meet the specific criteria for *IUCrJ* are transferred (with the contact author’s consent) for consideration by one of the other IUCr journals.

From 2014 until the pandemic, and well into 2021, the number of papers published in *IUCrJ* each year grew (with some fluctuation) from 70 papers in 2014 to 120 in 2019, 129 in 2020, and still 108 papers for the whole of 2021. Like many scientific journals elsewhere, by 2022 the hiatus in throughput of ongoing experimental research brought about by the pandemic, together with extended shutdowns of various X-ray and neutron facilities for upgrades and other issues around the world, resulted in a decline in submissions. However, we are pleased to report some promising signs of recovery – with 90 papers published in *IUCrJ* in 2024. As new or upgraded facilities return or come online over the next couple of years, we look forward to receiving many cutting-edge high-quality submissions to *IUCrJ*. Meanwhile, during the pandemic, prioritized handling was offered for papers on the SARS-CoV-2 (COVID) virus and this resulted in several well cited papers being published in *IUCrJ* (*e.g.* Michalska *et al.*, 2020 ▸; Kneller *et al.*, 2020 ▸; Melero *et al.*, 2020 ▸; Jaskolski *et al.*, 2021 ▸; Kneller *et al.*, 2021 ▸; Ebrahim *et al.*, 2022 ▸).

The relationship between *IUCrJ* and the other IUCr journals continues to evolve and mature. With online Special Issues, such as the present ongoing collection of Quantum Crystallography articles, involving more than one IUCr journal, papers in a Special Issue that meet the *IUCrJ* pre-selection requirements, determined by Main Editor vote, can appear in *IUCrJ* (*e.g.* Chodkiewicz *et al.*, 2024 ▸). Others in the Special Issue can establish the presence of new research areas in one or more of the other IUCr journals (all of course subject to normal IUCr journal review). Meanwhile, as open access increasingly becomes the norm for new and for a growing number of existing journals, the need for open data and curated databases has also grown. Most especially in crystallography and structural science, experimental results frequently comprise ever-larger datasets that need to be readily coordinated with large-scale computer simulations and models. Thus, *IUCrJ* is taking a role in carrying papers that seek to develop and define these links between cross-cutting high-impact structural science research and access to the underlying data and simulation results supporting the research results published in any IUCr journal (*e.g.* Guo *et al.*, 2020 ▸; Ferrence *et al.*, 2023 ▸; Brink *et al.*, 2024 ▸, Trewhella *et al.*, 2024 ▸).

The most significant technical development in the past few years, affecting all aspects of science and technology, is of course the emergence of machine learning (ML) and artificial intelligence (AI) tools. These are having major impact across all areas of structural science, most especially in protein crystallography, but more generally in any areas that depend on the successful curation and interrogation of increasingly large datasets, such as those collected at third- and fourth-generation synchrotron and free-electron laser facilities, or the newest generation of pulsed neutron source facilities. Over the next few years ML and AI methods will permeate much of the cutting-edge research reported in IUCr journals, including *IUCrJ*. We note that the Physics and Chemistry Nobel Prizes in 2024 were awarded for achievements associated with the development and application of AI and ML methods, and it is an honor to acknowledge one of the Chemistry Nobel Laureates, David Baker, for his two papers published in *IUCrJ* (Simkovic *et al.*, 2017 ▸; Farrell *et al.*, 2020 ▸), as well as multiple papers in other IUCr journals.

In closing, it is a pleasure to acknowledge Peter Strickland of the IUCr Journals office in Chester, UK. He has served as the *IUCrJ* Managing Editor from its launch in 2014 until his well deserved retirement in 2024. We now welcome Nicola Ashcroft, who has transferred from *Acta Cryst. A*, to succeed Peter as *IUCrJ*’s Managing Editor (Andrea Hill has succeeded Nicola at *Acta Cryst. A*). Meanwhile, Louise Jones has succeeded Peter as Head of Publishing Operations for all of the IUCr journals, and the Journals office will shortly welcome Kruna Vukmirovic as Head of Publishing Strategy. With Nicola Ashcroft as the new *IUCrJ* Managing Editor, together with the dedication of the *IUCrJ* Main Editors, Co-editors and Advisory Board Editors, *IUCrJ* now looks forward to an exciting future in its second decade.
